# Poverty and the re-growth of private renting in the UK, 1994-2018

**DOI:** 10.1371/journal.pone.0228273

**Published:** 2020-02-05

**Authors:** Nick Bailey

**Affiliations:** Urban Studies, University of Glasgow, Glasgow, United Kingdom; Cardiff University, UNITED KINGDOM

## Abstract

Over the last two decades, private renting has undergone a major revival in the UK, more than doubling its share within the housing system. Young adults increasingly remain in the sector into their 30s, giving rise to the term ‘Generation Rent’. Using data from the UK’s *Family Resources Survey*, this article shows how reliance on the sector varies by poverty status, particularly for young adults and children. In 2017/18, 42 per cent of adults under 40 in low-income poverty lived in private renting, compared with just 26 per cent of non-poor. This is almost double the proportion of 20 years earlier. Private renting is now home to more poor adults under 40 than owner occupation and social renting combined. In addition, one in three children in poverty (36 per cent) now lives in private renting, three times the level of 20 years ago. For both adults and children, rates are even higher in London and the South. Although rates of increase have slowed in recent years, this dramatic shift in the housing circumstances of those in poverty has a number of implications for housing and social policy which have not yet been sufficiently recognised.

## Introduction

The re-growth of the private rented sector (PRS) over the last two decades marks a fundamental change in the UK’s housing system. It is a trend which is apparent in a number of advanced industrialised countries, especially as access to homeownership has become more difficult [1] [2] [3], but it is particularly pronounced here, with the sector rising from 9 per cent of dwellings in 1991 to 19 per cent in 2017 (https://www.gov.uk/government/statistical-data-sets/live-tables-on-dwelling-stock-including-vacants). This re-growth has been at the expense of both owner occupation and social renting, and it has been very heavily focussed on younger adults. With increasing barriers to mortgage finance, stagnating earnings and a dwindling social housing supply, they can now expect to spend many more years in this sector than their parents, giving rise to the term ‘Generation Rent’ [4] [5].

In drawing attention to the divide between generations, however, this term serves to obscure important divisions within the current generation of young people and hence to mask the implications for social and housing policy. A number of studies have begun to reveal some of these divisions. Studies have shown that it is those young people who cannot draw on parental resources to help buy who will spend longer in private renting [6]. Kemp [7] shows that, even in 2007, the sector in England was playing a disproportionate role in housing those in low-income poverty and that this produced poorer outcomes for them in terms of housing standards and affordability. Coulter [8] shows how it is more disadvantaged groups in particular which saw rates of private renting rising between 2001 and 2011. In Northern Ireland, low-income households were making up an increasing share of the PRS [9].

To date, however, there has not been a systematic analysis of the changing role of the sector in relation to poverty. One possible reason for this omission is that such a change was never envisaged in UK policy efforts to revitalise the PRS. The overwhelming focus there has been on stemming the long-term decline of the sector in order to maintain flexibility in the housing system. The PRS is the sector best placed to provide rapid access to meet short-term housing needs. As such it provides housing for young, mobile workers in particular, aiding the functioning of local economies. It was never promoted as a medium- to long-term housing solution for low income households because of its obvious shortcomings in this regard. Compared with the main alternative of social renting, it is characterised by much greater problems of affordability, lower property and management standards, and greater insecurity [7] [8] [10]. The sector seems particularly ill-suited to meet the needs of low-income families where frequent movement might be expected to be harmful to children’s social and educational development.

The aim of this article is to examine the changing role of the PRS in housing young adults and children living in poverty. The article uses data from a UK household survey designed to provide rich detail on household financial circumstances, the *Family Resources Survey* (FRS). This is a continuous cross-sectional household survey, covering a sample of 1.3 million individuals over the years 1994/95-2017/18. It is this data which the UK Government uses for its annual *Households Below Average Income* (HBAI) analysis [11]–effectively the official report on poverty in the UK. A particular novelty of the analysis is the use of Lexis surface visualisations [12] to capture trends over time and by age in great detail. These enrich our understanding of the changes in a way which is immediately accessible, providing a clearer understanding of how tenure change is occurring and who is affected.

In the remainder of this introduction, the background to the re-growth of the PRS is examined in more detail. The article then discusses the data and analytical approach. Findings cover the shifts in housing tenure first, before looking at the situations of adults and children separately, broken down by poverty status. The article also examines regional variations and whether the experience of insecurity within the PRS varies with poverty status. It concludes with a discussion of the issues raised for social and housing policy.

### Poverty and the PRS

For most of the last century, private renting in the UK was in decline. Its role in housing those in poverty became very marginal as the UK developed a substantial social rented sector with a strong welfare focus [13] [14] [15] [16]. The term ‘social rented sector’ covers a range of organisations providing rental housing at below-market rents, usually relying in part on provider subsidies from national government which also regulate their activities to a high degree. Social landlords include public bodies, notably local authorities, as well as those in the voluntary or third sector, mostly termed housing associations. Social tenants usually have security of tenure i.e. the right to remain in the dwelling as long as they want provided they meet the terms of the lease. Dwellings are allocated on the basis of social and housing need, and tenancies cannot usually be passed on or inherited.

Compared with other countries with liberal welfare regimes, the UK was unique in having such a substantial social rented sector. In the US, Canada, Australia and New Zealand, social renting accounts for around 5 per cent of the housing stock [17]. In the UK, social renting peaked at 33 per cent in 1981 and, even after nearly four decades of decline, it remains at 18 per cent in 2017 (https://www.gov.uk/government/statistical-data-sets/live-tables-on-dwelling-stock-including-vacants). As social landlords focussed on meeting housing needs and these needs are strongly related to poverty [18], the social rented sector housed a substantial proportion of low-income households. It ensured they were able to achieve decent, secure housing despite the relatively low levels of means-tested welfare benefits in the UK [19]. The sector declined in size after 1981, driven by a shift in policy from social provision to commodified market provision, common across many countries [1, 5, 14]. As a result, it became even more closely associated with social need, in part through the selective loss of better-off households through the Right-to-Buy and in part through the increasing focus on meeting housing need under homeless persons’ legislation [20].

Home ownership, by contrast, has long been seen as the aspirational tenure and promoted as such by successive UK Governments [21], but it too has come to play an expanding role in housing those in poverty. From the 1980s, its growth was fuelled by increasing numbers of lower income households, encouraged by government policy which helped ‘normalise’ ownership [5, 21]. Some entered through the heavily-discounted sales of council housing to sitting tenants under the Right-to-Buy while others on low and uncertain incomes found they could access mortgage finance as the deregulation of the mortgage market fuelled intense competition between lenders. Burrows [22] showed that, with a broad poverty measure, half the poor were homeowners in 1999 although with narrower measures, the proportion was rather lower. Similarly, Wallace [23] showed that half of all people in low-income poverty on the ‘before housing cost’ measure were owners in 2013/14, although this share fell to less than one third on the ‘after housing cost’ measure. Nevertheless, the broad message that a substantial proportion of the poor are home owners became widely recognised. Similar shifts occurred in many countries with the consequences all too apparent in the wave of repossessions following the housing market crash of 2007/8 [1].

From the 1980s, a political consensus emerged in the UK on the need to halt the decline of private renting [24]. In public discourse at least, this was driven almost entirely by concern over its role in providing short-term, flexible housing for young, mobile workers and hence aiding local economic functioning although this approach fits also with the longer-term shift towards commodification in housing noted above. It was recognised that the sector might continue to play a minor role for low-income households, notably those regarded as a low priority for social housing [25] but this was seen very much as a ‘residual’ role for the PRS because of the assumed preference of these households for social renting. More generally, the sector was seen as a transitional or ‘waiting room’ tenure [25] accommodating people for a relatively short period before they moved on to one of the two dominant tenures.

The role of the PRS in relation to poverty was also seen as residual in a second sense: as Kemp [7] argues, the PRS is “a demonstrably inferior tenure for low-income households” (p1020). The high costs of private rents can act as a barrier to employment or push working households into poverty [10]. Poor PRS tenants are almost twice as likely to find it difficult to pay their rent as poor social tenants [7]. In addition, the PRS has long been noted for its poor property standards and poor quality of management. As it has expanded recently, this has raised the average quality of properties but those in the PRS remain more likely to have substandard housing [7]. Poor PRS tenants are as likely to struggle to keep their home warm in winter as poor social tenants, and both are worse off than poor owners and non-poor renters [7].

Most importantly, perhaps, private renting in the UK, as in many other liberal welfare regimes, is distinguished by high levels of insecurity, particularly in comparison with social renting [26]. This insecurity was the direct goal of deregulation policies in the late 1980s as these were seen as a necessary pre-condition for attracting re-investment [27] [28]. It is potentially a more significant problem for lower income households for two reasons. First, frequent moves may disrupt social connections to family and friends which low-income households are particularly reliant on as a source of practical and emotional support [29]. These connections are especially important for those with children, and lone parents most of all [30]. For children, there is the additional concern that frequent moves may disrupt not only their social networks but also their schooling, with longer term impacts on educational attainment. Second, the subjective experience of insecurity [31] may be more problematic for those in poverty. As their housing options are much more limited, the threat of loss of accommodation is likely to be a particular cause of stress.

While some aspects of housing policy in the UK are devolved to regional governments, devolution has very limited relevance here [13] [14] [15]. In terms of policy, one key difference noted already is that Scottish local authorities pursued development of social housing in the postwar period to a much greater extent than those in the rest of the UK. In other devolved policy areas, there has very largely been parallel development. For example, Right-to-Buy policies were very closely aligned for most of the last 35 years although there has been some divergence very recently and, with security of tenure for the PRS, near-identical legal reforms were enacted in 1988. Scotland may very recently have moved to end ‘no-fault’ evictions but that happened too late to have any bearing on the picture here and may in any case prove more symbolic than substantive. Beyond explicit housing policy, the factors which have driven changes in the housing system are UK-wide. The UK government sets tax policies impacting on incentives to own or the returns from private renting. Pensions policy reforms which provided further incentives for investment in private renting were also UK-wide as were the impacts of the GFC on mortgage lending criteria. Variations in housing affordability appear to be important but these operate across the UK, not just of devolved regions, and are discussed on that basis.

### Welfare reform and the PRS

In the UK, the ability of low-income households to access private renting and the quality of the accommodation they can afford depends very largely on the means-tested Local Housing Allowance (LHA)–as noted above, a part of the Housing Benefit system which also covers households in social renting but with slightly differing entitlements [32]. Around one-in-four PRS households claims LHA. From April 2011, the Government sought to limit expenditure on LHA by: reducing the maximum amount of rent for which LHA could be claimed from the median rent for properties in the local area to the 30^th^ centile; placing an absolute maximum cap on LHA by size of property regardless of location; and restricting claims from under 35s to the rent for a single room (previously a restriction only applied to under 25s). From 2013/14, the Government limited increases in these allowances to the Consumer Prices Index, cutting this again to a maximum of 1 per cent from 2014/15 [33]

These changes were achieving annual savings of £1.7 billion by March 2016, impacting on 1.4 million households [34]. Surveys of private landlords in 2015/16 and 2017 suggest that large proportions are becoming less willing to let to people on LHA (or its successor, Universal Credit) because of these changes and that many are demanding additional safeguards (rent guarantors and/or higher deposits) or raising rents in response [35] [36]. Results of the broader evaluation on the impacts of LHA reforms on tenants and landlords are available from the Department for Work and Pensions website (https://www.gov.uk/government/publications/local-housing-allowance-monitoring-the-impact-of-changes [Accessed 1 December 2019]).

These changes can be seen as part of a wider process of restructuring welfare in the UK, which has been underway for some time but given additional impetus by the Global Financial Crisis (GFC) [1] [4] [21]. In the UK, one specific aspect of this restructuring has been the shift in welfare support away from working age groups towards the elderly. Pensioners saw poverty rates halve in the 2000s [11], thanks to additional financial support from the New Labour government. The value of these benefits has been protected from ‘austerity’ so that the burden of that policy has fallen substantially on the working age population. As a result, poverty has shifted to working age adults and children: from 80 per cent of the poor in 1996/97, they make up 87 per cent in 2017/18 [11]. Young adults have faced a particularly harsh time, as the example of LHA reductions makes clear, becoming more dependent for their welfare on their family [37].

### Regional variations

The re-growth of the PRS has occurred across the UK but its role in relation to poverty is likely to vary between regions, reflecting the relative accessibility of other tenures in any given area. The affordability of owner occupation varies widely, with London and the South facing far higher costs relative to local wages compared with the Midlands and the North [38]. The supply of social housing also varies. Scotland has had historically high levels although the gap with the rest of the UK is narrowing, while London also has a relatively large sector. Bramley [18] shows how these variations impact on a range of housing outcomes, with higher levels of unmet need in London and the South, compared with the Midlands and the North. It is the same areas which face the greatest impacts from the restrictions on LHA: one third of the affected households are in London, and a further third in the rest of the South [34].

### Concealed households and other sharing arrangements

Analyses of changes in tenure are often presented on the basis of households but this masks the position of people living as a member of someone else’s household [18]. This category covers many young adults who may not wish to leave home but it also includes others (and their dependents) who are unable to achieve independence. There tends to be a divide here by social status [39]; young adults with lower educational attainment tend to leave education earlier, entering the labour market and forming independent households at a younger age. In many EU countries, delaying the move to independence has been a more common response to problems in accessing home ownership than a move to private renting, but the UK has also seen rising proportions of young adults remaining at home [2].

### Summary

The aim of the paper is to assess how the role of the PRS in relation to people in poverty has changed over the last two decades. It looks at the whole of the UK over a period of 24 years and seeks to identify how these changes vary by age, for both adults and children. There is a particular focus on the most recent years when access to LHA has become more restricted. It also explores variations across regions in relation to housing affordability. Lastly, given the importance of insecurity of tenure in discussions about private renting, the paper also examines relative levels of instability in the PRS, and how these vary by poverty status.

## Data and methods

### Data

Data are taken from the continuous cross-sectional household survey, the *Family Resources Survey* (FRS), for the years 1994/95-2017/18 [40], merged with the associated *Households Below Average Income* (HBAI) data for the same period [41]. The FRS is an annual UK government survey which captures detailed measures of household resources. It provides data on the household including household composition, housing tenure and total income, as well as data on each adult and child within the household including age, householder status and, if not the householder, relationship to the householder(s). Children are defined as all those below 16, or 16–18 (occasionally 19) and still in full-time education. The HBAI provides additional derived household-level variables, including the official measures of low-income poverty, child material deprivation and severe child poverty (see below and [41] for the technical details). There is a unique key which enables records to be matched exactly between the two datasets. The HBAI data also include a set of grossing weights to ensure the data match known age/sex distributions at regional level, and these are used throughout the analyses here. Across the 24 years covered, the unweighted data provide information on 1.34 million people–1,010,000 adults and 325,000 children.

### Analysis

As noted above, we are interested in tenure for individuals. The FRS identifies the three main tenure groups: owner occupiers, social renters and private renters. The last includes those who occupy their accommodation by virtue of their employment (tied accommodation). In addition, we identify adults within the household who are neither the householder (tenant/owner) nor the partner of the householder. We classify all of these as living ‘care of’ their household as they do not have householder status. On average, 12 per cent of adults are in this situation, a figure which has remained quite static over the last 24 years. The majority are the daughter/son of the householder or of their partner. In addition, a small number of people identify themselves as living ‘rent free’ (around 1 per cent) as they occupy a dwelling typically owned by a family member or friends without paying rent. They are included in this group on the basis that they have not shown the ability to become householders in one of the conventional tenures. We therefore allocate adults to one of four tenures: owner occupation (including shared ownership); social renting; private renting (including tied accommodation); and ‘care of/rent free’. Children are allocated the tenure of their household so cannot be regarded as ‘care of’; there are around 1 per cent of children where their parent is not the householder, i.e. they are part of a concealed household.

The main poverty measure used in the paper is the relative low-income poverty measure, widely used in poverty analysis including by UK Government. It identifies households with an equivalised income below 60 per cent of the contemporary median; full details are provided in [11] and [40]. Like Kemp [7], we favour the ‘after housing costs’ (AHC) measure since the high costs of private renting are a central part of the problem faced by lower income households in the sector. Results using the BHC measure show similar trends and are made available through the on-line visualiser (details below).

Since 2004, the Government has used the FRS to measure material deprivation for children as part of the UK’s official measure of child poverty [42]. Information is collected on a set of 21 items which are regarded as ‘necessities’ by a majority of the UK population, i.e. things which everyone should be able to afford to have or do, and no one should have to go without. Items cover both household and child consumption. For the household, they include things such as the ability to keep the home warm in winter or to pay for home insurance. For children, they cover things such as a warm coat for winter or money to take part in school trips or activities. Households with dependent children are asked whether they or their children lack each of these items and, if so, whether that is due to being unable to afford them (rather than not wanting them). The Government uses a system of prevalence weighting to create a combined score for the 21 items which ranges from 0 to 100, with 25 or more used to identify ‘material deprivation’. The set of items was updated by the Government in 2011 [42] creating some minor discontinuity in the measure. For additional background on the development of these measures and the current methodology see Bailey and Bramley [43].

Two further poverty measures are available for households with children, identifying those with both low income and material deprivation on the basis of two different low-income thresholds. For contrast, this paper uses the more extreme version, where households have an income below 50 per cent of the contemporary median after housing costs as well as material deprivation–a situation termed by the Government ‘severe poverty’. In 2017/18, 29 per cent of children were in low-income poverty, but only 18 per cent were materially deprived and just 5 per cent were in severe poverty.

To examine regional variations, we group the UK’s standard regions on the basis of a measure of housing affordability produced by the Office for National Statistics (ONS) [38]: the ratio of median house prices to median gross annual earnings. London stands out for its poor affordability ratio (12.9 in 2016), followed by the three Southern regions (8.3 to 9.5), the Midlands (6.2 to 6.4) and the North (5.1 to 5.7). Wales (5.7) is included with the North. Scotland (not covered by the ONS publication) is treated separately as is Northern Ireland which is only included in the FRS from 2002/3 onwards.

For people in the PRS, there are no measures of the household’s legal tenure security on the FRS, i.e. the precise legal arrangement under which they hold their tenancy. We cannot distinguish those with some measure of security from those on short-term, insecure tenancies which are the norm. Instead, to get some measure of security in practice, we look at patterns of recent mobility: the proportion of households resident in their dwelling for less than one year. The FRS asks the householder how long they have lived in the property and this is used to provide a single measure for all household members. The way in which the question is asked changed slightly from 2012 onwards, and this creates a modest discontinuity in the data.

Methodologically, the paper makes use of Lexis surfaces. These visualisations developed within demography as a means of portraying the complex interactions of age-period-cohort effects on mortality or morbidity [12]. Here they are used to show how people are distributed across tenures, at different ages and time points, and for different sub-groups such as the poor. A modest amount of smoothing is applied to reduce noise and preserve anonymity: the value for each cell is averaged with those for the adjacent four cells horizontally and vertically. A red-green-blue colour scheme is used to represent percentages. As this may not be accessible to some people with colour-vision impairment, an on-line visualisation tool has been set up (https://ubdc-apps.shinyapps.io/data_explorer_adult/) to allow users to experiment with a wide range of colour schemes. Users can also explore how the choice of poverty measure (before or after housing costs) influences results and examine variations between regions in much more detail.

### Data access and reproducibility

Individual-level survey data were obtained from the UK Data Service (UKDS), and are up-to-date as at July 2019. The UKDS provides technical reports on the FRS and HBAI datasets. The SPSS code used to produce this analysis has been made available through Github (https://github.com/nick-bailey/Poverty-and-private-renting) so others who register for these data can reproduce the analyses presented here. The aggregated data used to create the Lexis surface visualisations and the line charts can be downloaded from the same place.

## Results

### Tenure change

**Fig 1:**
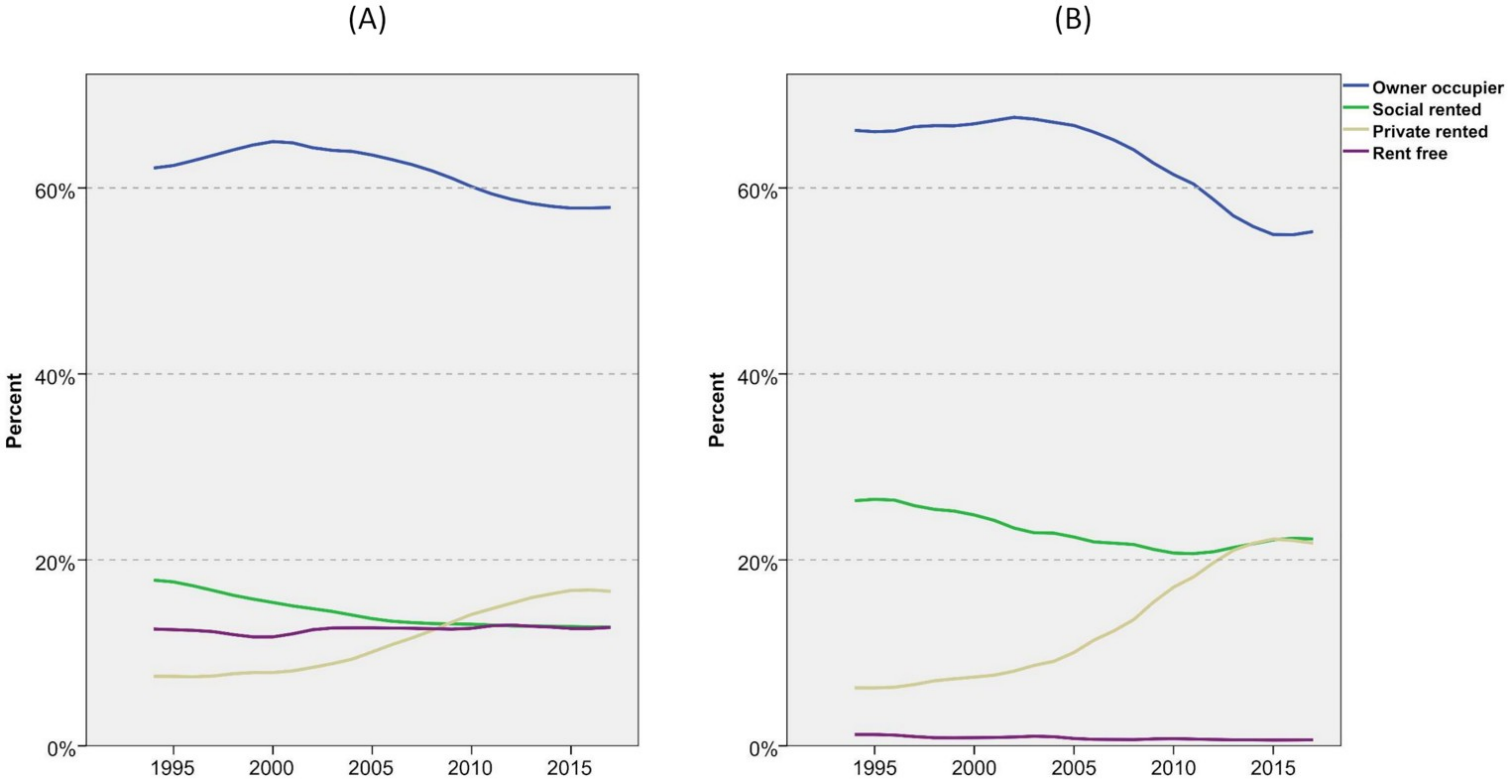
Tenure for adults and children, 1994/5-2017/8. (A) Adults. (B) Children. FRS/HBAI data. Years are financial years (i.e. ‘2017’ is 2017/18).

Fig 1 shows tenure change in the UK over the last 24 years for adults (A) and children (B). For adults, home ownership peaks around 2000, since when it has declined at a steady rate while social renting declines through most of this period, due largely to the Right-to-Buy. Only private renting shows growth, up from 8 per cent in 1994/95 to 17 per cent in 2017/18, albeit with a noticeable slowing in the last few years. The proportion of adults who are not householders has remained quite steady at around 12 or 13 per cent for the whole period. We do not see strong evidence of young adults remaining longer in the family home in these data, at least when considering adults as a whole.

For children, the changes in tenure are even more dramatic. The proportion in owner occupied households has fallen much more sharply since the GFC as families have found it particularly difficult to access mortgage finance although as previously, this decline appears to have halted in the last couple of years. There are now as many children in private rented households as in social rented ones which represents a fundamental shift in the UK housing system. Private renting is up from one-in-twenty children in 1994/95 to more than one-in-five in 2017/18 (6 to 22 per cent) although, again, the rate of growth appears to have slowed in the last few years. The proportion of children in social renting has declined over the period as a whole although it has shown modest recovery in the last five years. As the overall size of social renting has not risen, the sector is gradually reorienting to play a greater role in meeting the needs of low-income families.

**Fig 2:**
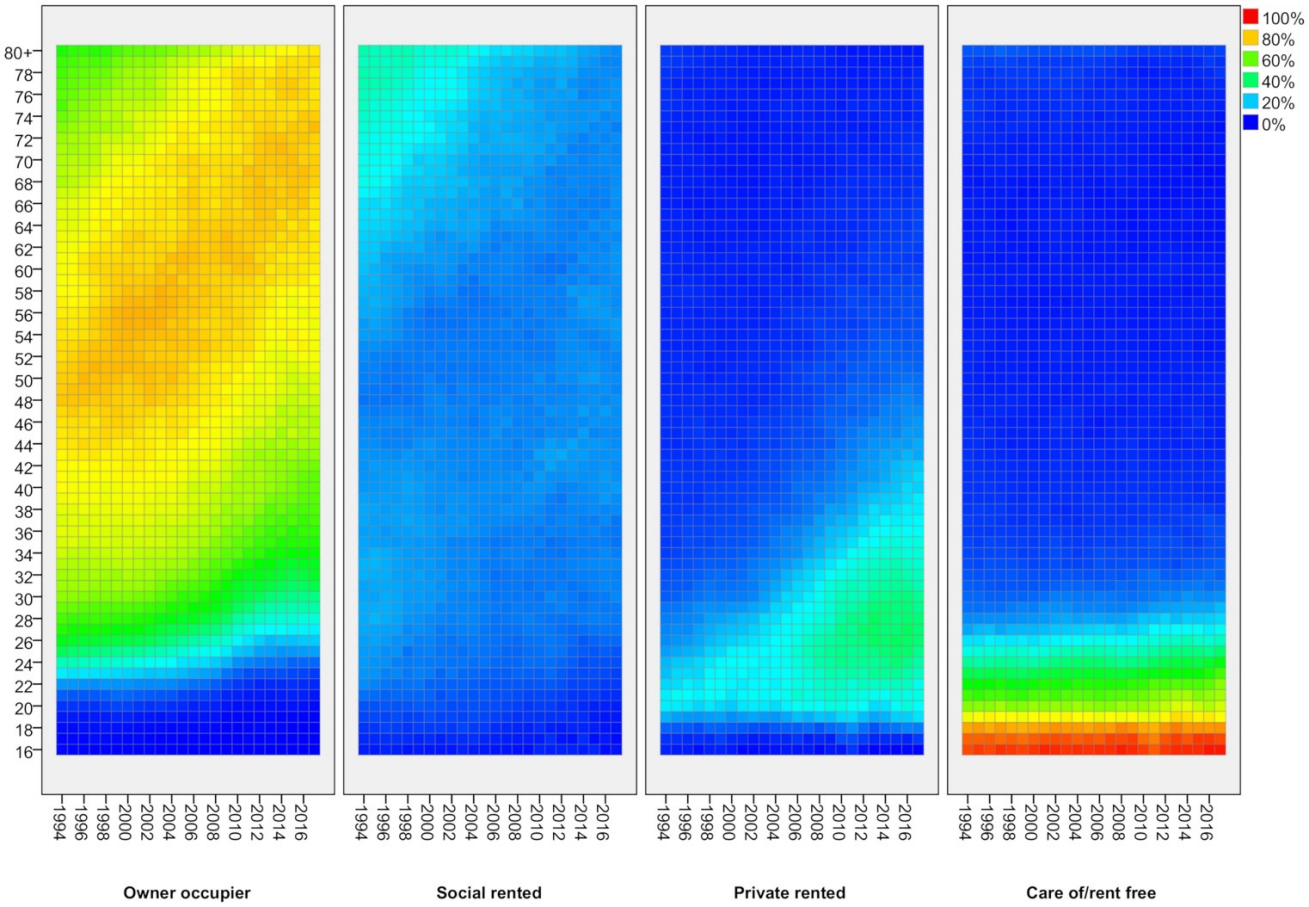
Tenure by age—Adults, 1994/5-2017/8. FRS/HBAI data. Tenure based on individual adults. Years are financial years (i.e. ‘2017’ is 2017/18). In each cell, the colour represents the proportion of people of that age in that year in a given tenure.

**Fig 3:**
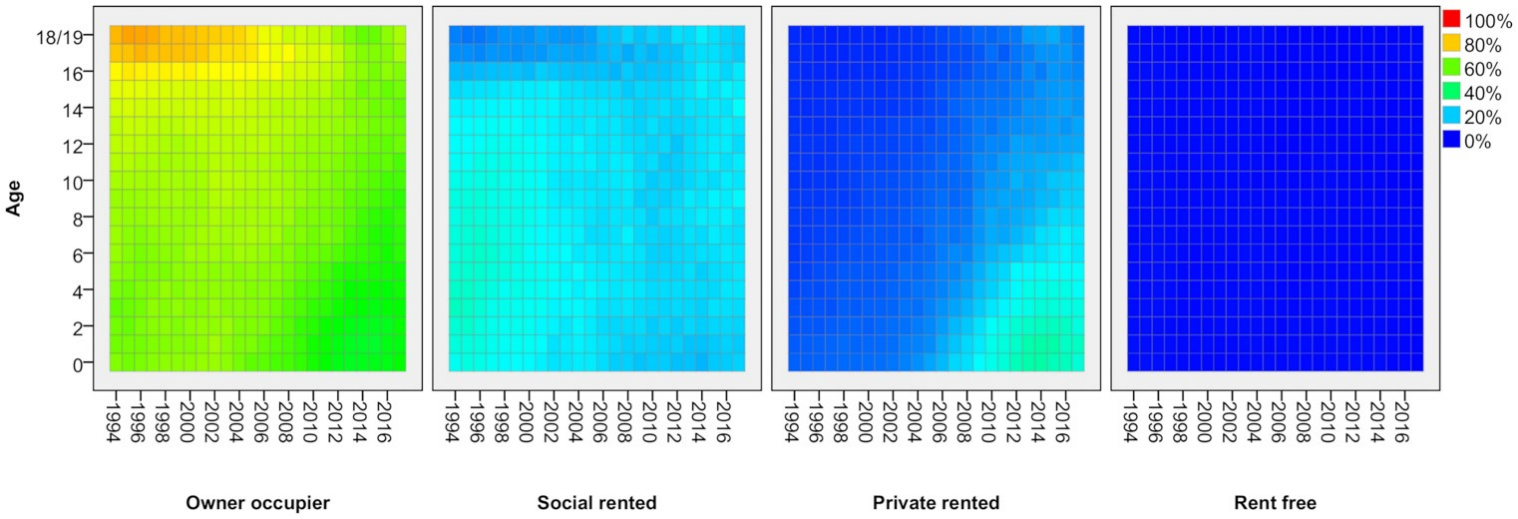
Tenure by age—Children, 1994/5-2017/8. FRS/HBAI data. Tenure based on householder. Years are financial years (i.e. ‘2017’ is 2017/18). In each cell, the colour represents the proportion of people of that age in that year in a given tenure.

Lexis surfaces reveal much greater detail for these changes by age, for adults (Fig 2) and children (Fig 3). In effect, each line in Fig 1 shows the average proportion across all age groups in a given tenure at each point in time. With the Lexis surface, each line is expanded into a pane which shows the proportion in that tenure separately for every single year of age (vertical axis) and how that has changed over time (horizontal axis). Each cell shows the proportion in a given tenure for people of a particular age in a particular year. Blue represents lower proportions in a given tenure, moving through green to orange and red for the highest proportions. As people age one year with each year of time that passes, each cohort tracks diagonally up to the right over time (though note that, as these are not longitudinal data, it is not the same individuals being tracked but measurement through repeated cross-sections).

The value of Lexis surfaces is that they reveal in much greater detail how a given social process impacts differentially across the life-course and they provide strong indications of the kinds of factor at work. Where there is a change which impacts all age groups at a particular point in time, the surfaces show a strong vertical feature–a period effect, in the language of demography [12]. There are no such effects evident here which is not surprising given how durable housing tenure is as a social status. Where there is a change which affects people of the same age consistently over time, we see strong horizontal features or age effects. For adults, we see an example in the fourth pane of Fig 2 which shows how all young adults going through the same process of leaving the family home through late teens and early twenties and, to a lesser extent in the third pane, entering private renting. Lastly, where there is a change which affects a specific cohort of people, we see strong diagonal features. These are effects which start to affect some age groups at a point in time but which then persist for those age groups so that, in each subsequent year, their situation is different to that for the equivalent age group in previous years. There are several strong diagonal features in these charts, notably in relation to private renting for adults and children.

The first panel in Fig 2 shows the proportion of adults who are owner occupiers (for any given age/year). It shows how people tend to attain the status of home owners through their working age years, but it also shows the ageing profile of home owners over the period covered here. Around 2000 when home ownership rates were highest, the peak age for ownership was in the 50s. At that time, home ownership rates for people of retirement age were lower as fewer had made it to home ownership during their working lives in earlier periods. As barriers to entry to home ownership rose after 2007/8, people are taking longer to achieve ownership and fewer are doing so. Ownership rates are dropping for all working age groups so the peak ownership group is getting older. This upward shift does appear to have halted in the last few years, however.

Social renting (second panel) has the most neutral age distribution now. In the 1990s, it housed disproportionate numbers of pensioners but that is no longer the case. We can also see some signs of the slowing of entry to this tenure for young adults in their 20s, reflecting the declining availability of social housing. By contrast, the third panel shows very clearly how the expansion of private renting is strongly concentrated among young adults. The green triangle bottom-right in that panel represents ‘Generation Rent’ in a simple visual form. This appears to be a classic ‘cohort effect’–a change that affects those who were 20 or younger around 2000 and which has remained a feature for this group as they have aged. Before 2000, the sector was focussed largely on those in their early 20s but they would typically spend only a few years in it before moving on to one of the two dominant tenures. By 2015, the PRS was home to a significant proportion of those in their 20s and mid-30s, and they can expect to spend more years in the sector on average.

The last panel shows the proportion of people living ‘care of’ (or in a small minority of cases, ‘rent free’). Almost all 16–19 year olds live ‘care of’ their parents, moving out during their twenties. There are some signs on an increase in the age of leaving home for this group over the period but it appears very modest when compared with the rise of the PRS for younger adults.

With transitions to owner occupation and social renting delayed, more young adults are starting families while renting privately, and this shows up in a rise in the proportion of children in the PRS, particularly young children (Fig 3, third panel). In 1994/5, just 8 per cent of children five or under were found in this sector. By 2017/18, it was 28 per cent. The proportion falls as children age, suggesting that most have left the PRS before they have been at school for more than a year or two.

One other prominent feature of Fig 3 is the tenure for children 17 and over, where owner occupation plays a disproportionate role in the years up to about 2008. This does not reflect the movement of households with older children into home ownership at this time. Rather, it reflects the much greater propensity for young people in these households to stay on at school after 16 at that time. In the late 1990s/early 2000s, those in social renting were much more likely to leave school so they were then counted as adults, effectively disappearing from the picture provided by this Figure. In the late 1990s, two-thirds of 17/18 years olds in owner-occupier households remained in school compared with just one third of those in social renting. The gap narrowed steadily until, from 2012, there was no difference and this feature therefore disappears.

### Adult poverty by tenure

**Fig 4:**
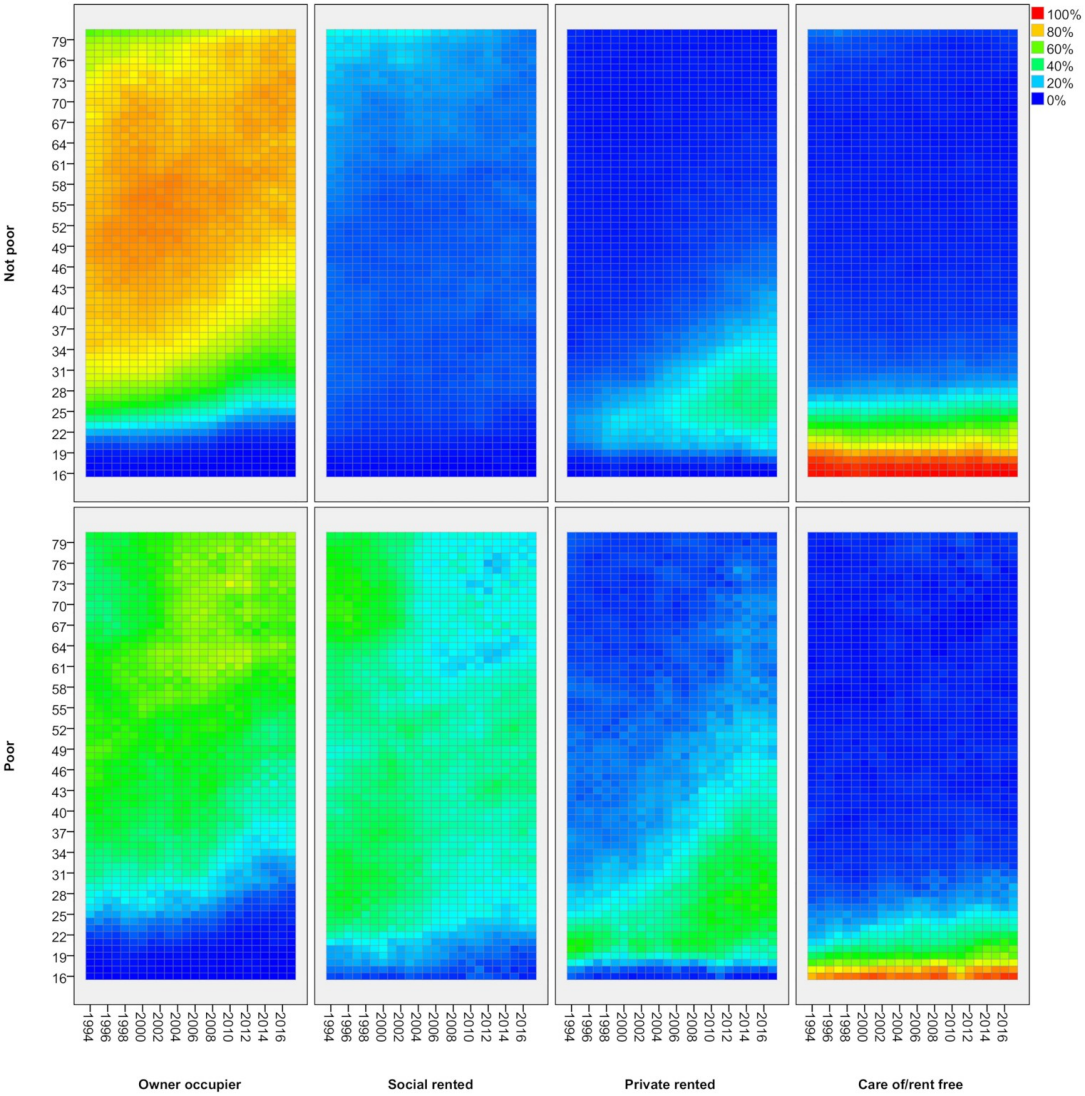
Tenure by age and poverty status—Adults, 1994/5-2017/8. FRS/HBAI data. Poverty defined as households below 60 per cent of contemporary median income, after housing costs. Years are financial years (i.e. ‘2017’ is 2017/18). In each cell, the colour represents the proportion of people of that age in that year in a given tenure, separately for poor and non-poor groups.

Fig 4 shows tenure changes for adults, now split between poor and non-poor groups using the relative low-income poverty measure (after housing costs). Colours now show, for a each age/year, the proportion in a given tenure within each poverty category. The Figure shows clearly the effective targeting of social housing on those in social needs. It houses relatively few non-poor households but a large share of the poor. In recent years, it has played less of a role in housing poor pensioners, more of whom are now found in home ownership. Owner occupation is clearly the dominant tenure for non-poor adults although the figure also shows how it house a substantial proportion of the (older) poor as well. The reductions in home ownership rates for working age groups since 2007 is apparent for both poor and non-poor but the drop is particularly marked for poor adults in their 30s/early 40s.

Fig 4 also shows the clear divisions within ‘Generation Rent’. For poor adults, the growth in private renting is far greater and reaches well into their 30s and 40s for a substantial proportion. Indeed, the share of young adults in private renting is now greater than the share in social renting: the PRS has replaced social renting as the home for those who are young and poor. For non-poor adults, private renting is more largely confined to those in their 20s/early 30s. Their exit to owner occupation is later than it was twenty years ago but few remain renting privately after about 30.

The Figure also shows how housing pathways for the youngest adults depend on poverty status as discussed above. Those in non-poor households tend to remain longer in the family home as they continue for longer in education. Those from poorer households tend to follow the ‘fast track’ route to independent living. For the former, there appears to be little change in the age at which they exit the family home although their destination now is much more likely to be private renting. For those in poor households, there is more evidence of a delayed departure and more now go to private renting rather than social.

**Fig 5:**
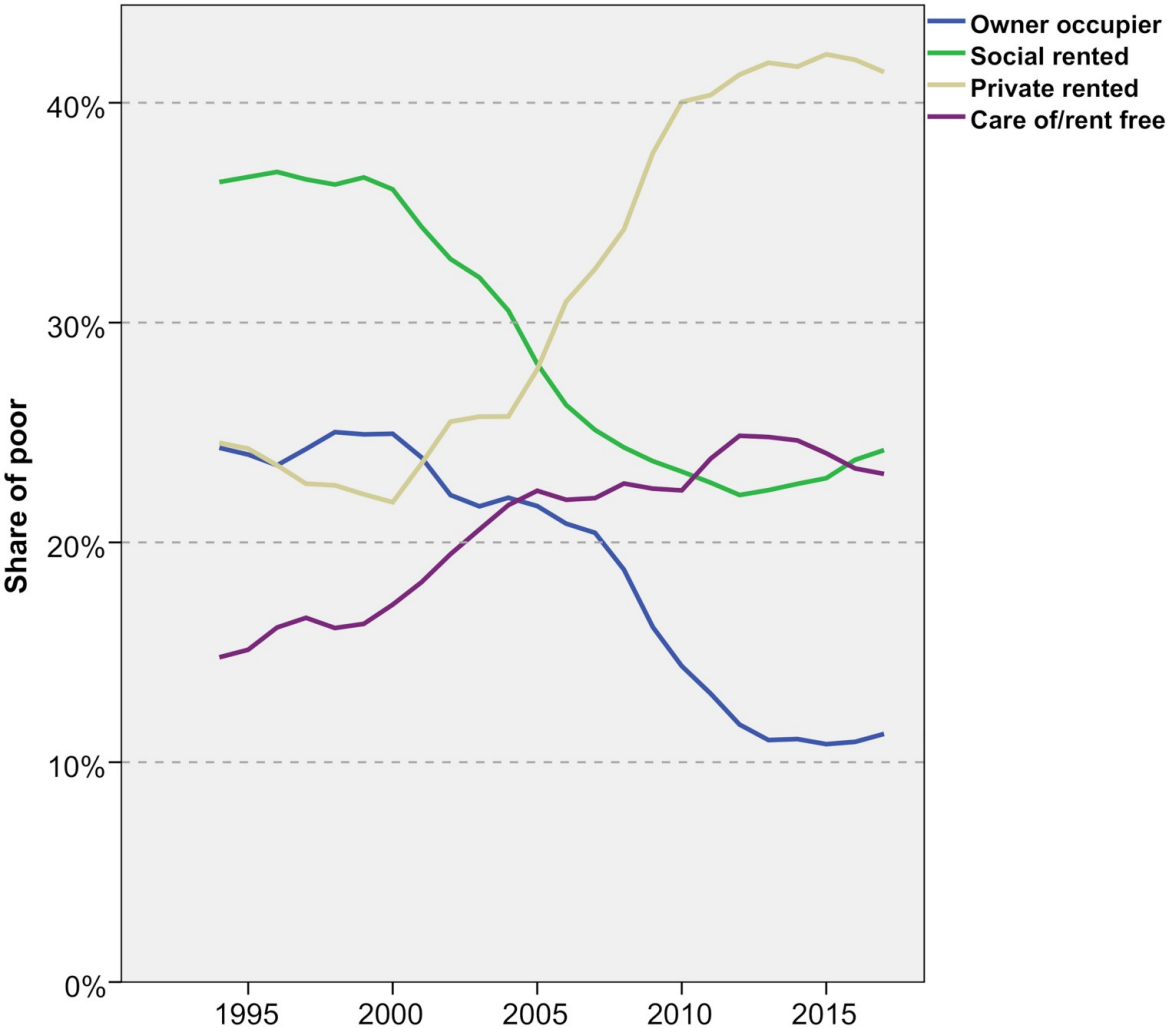
Share of poor adults under 40 by tenure—1994/5-2017/8. FRS/HBAI data. Poverty defined as households below 60 per cent of contemporary median income, after housing costs. Years are financial years (i.e. ‘2017’ is 2017/18).

Using the insights from the Lexis surfaces, Fig 5 summarises the trends for adults under 40, showing the share of poverty in each tenure. It shows very clearly the meteoric rise in the proportion of these people living in private renting between 2000 and 2010 as well as the more modest increase in the proportion living ‘care of’ another household. These two tenures now account for two thirds of all under 40s in poverty, up from just over one third 20 years ago. Private renting on its own is now home to 42 per cent of poor young adults, more than owner occupation and social renting combined.

One other important feature of Fig 5 is the marked slowing in the growth of private renting since about 20120, coinciding the changes in LHA eligibility. Indeed, there has been no increase over the last four years. We noted above the evidence that landlords were becoming more reluctant to let to those on LHA, and this Figure appears to support that. There was initially some rise in the proportion ‘care of’ in this period but that has fallen back. There has also been a halt to the decline of owner occupation and a modest rise in social renting.

**Fig 6:**
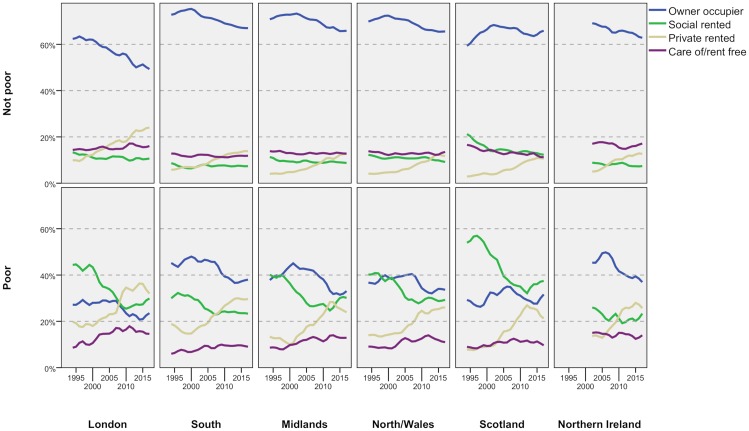
Regional variations in tenure by poverty status—Adults, 1994/5-2017/8. FRS/HBAI data. Poverty defined as households below 60 per cent of contemporary median income, after housing costs. Years are financial years (i.e. ‘2017’ is 2017/18). Data for Northern Ireland not available before 2002/3.

The rise in private renting has not been even across the country but has been particularly strong in the regions where high prices make buying less affordable, notably London and the South. In those cases, the proportion of poor adults in private renting is correspondingly higher (Fig 6). In London, the majority of poor adults in their late 20s/early 30s now live in the PRS and a large minority will rent privately through their 30s/early 40s. The proportions are slightly lower in the South and lower again in the rest of the country. In the Midlands and Scotland, the proportion has been falling in the last few years, largely because the proportion in social renting has been rising. The rises have halted or slowed in every other region as well.

Fig 1 showed that the proportion of adults living ‘care of’ another household had not risen overall. In Fig 6, we see that this masks rather different trends for poor and non-poor adults. For the non-poor, the share living ‘care of’ is fairly static or declining in every region whereas, for the poor, it is rising in every case except Northern Ireland. That region has a relatively high proportion to start with–only London is higher.

Fig 6 also shows that the slowing in the growth of private renting in the last five years applies to poor adults, not those who are not poor. In all the regions, the growth in private renting for non-poor has continued unabated, albeit at a lower absolute level, whereas the rise of poor private renters has either halted or reversed (with the possible exception of North/Wales). Again, this strongly suggests that the cuts in LHA have had a significant impact on landlords’ willingness to rent to low-income households although the improving access to social renting may also be a factor.

### Child poverty by tenure

**Fig 7:**
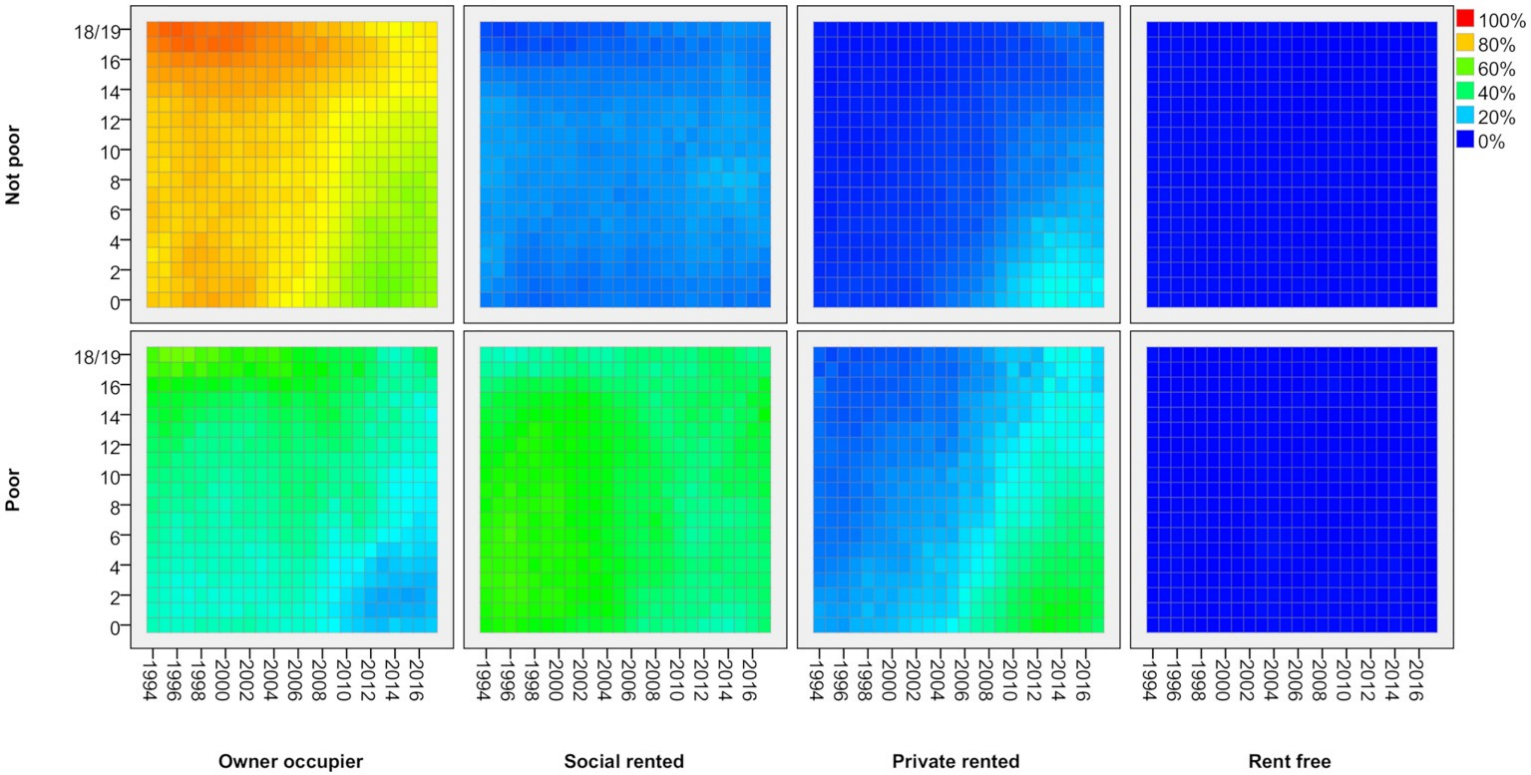
Tenure by age and poverty status—Children, 1994/5-2017/8. FRS/HBAI data. Poverty defined as households below 60 per cent of contemporary median income, after housing costs. Years are financial years (i.e. ‘2017’ is 2017/18).

Fig 7 shows the equivalent picture of tenure change for children by poverty status as for adults. The rise in private renting for those in poverty is far greater than for the rest, again revealing the divisions within Generation Rent. For non-poor children, the experience of private renting is limited to a small minority and confined very largely to the pre-school years. For poor children, the experience is both more common and likely to be longer lasting. Significant proportions of poor children are found in the PRS through primary and into secondary school years.

**Fig 8:**
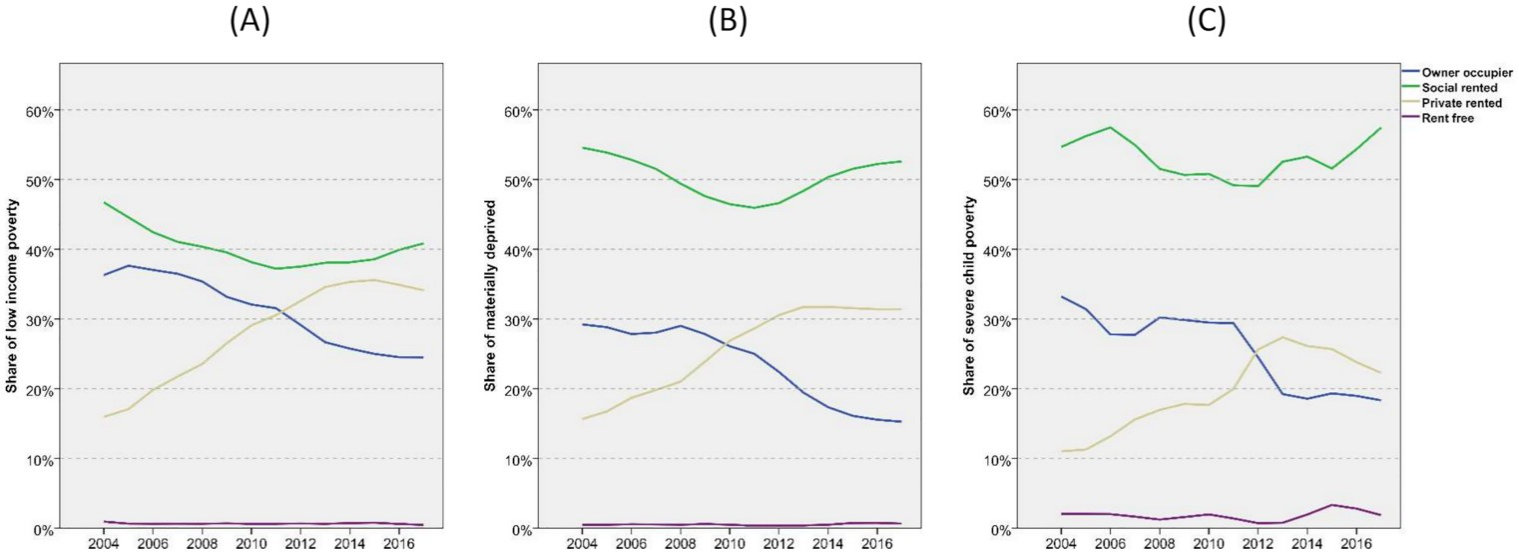
Tenure shares for children in low income poverty, material deprivation and severe poverty by tenure—2004/5-2017/8. (A) Children in low income poverty. (B) Children in material deprivation. (C) Children in severe child poverty. FRS/HBAI data. For definitions of material deprivation and severe child poverty see text and [11]. Years are financial years (i.e. ‘2017’ is 2017/18). Modest smoothing applied to data to limit noise.

For the years since 2004/5, the FRS provides two further measures of child poverty; see methods section above for details. These capture poverty at more extreme levels than the low-income measure used in the rest of this paper. All three show a consistent picture of rising levels of poverty in private renting and at similar rates but, with the more ‘extreme’ measures, the shares are slightly lower (Fig 8). The proportion of materially deprived children in the PRS has risen from 17 to 32 per cent in 12 years (central panel) while the proportion in severe child poverty has risen from 11 to 26 per cent (right panel). In all three cases, the PRS has overtaken owner occupation in the share of poor children it accommodates. Social renting plays a more important role for groups facing higher levels of disadvantage, and still houses half of those materially deprived or in severe poverty. Whichever measure we use, however, the growth of poverty in private renting has halted since 2013 while the shares in social renting have stopped falling and even begun to rise again.

**Fig 9:**
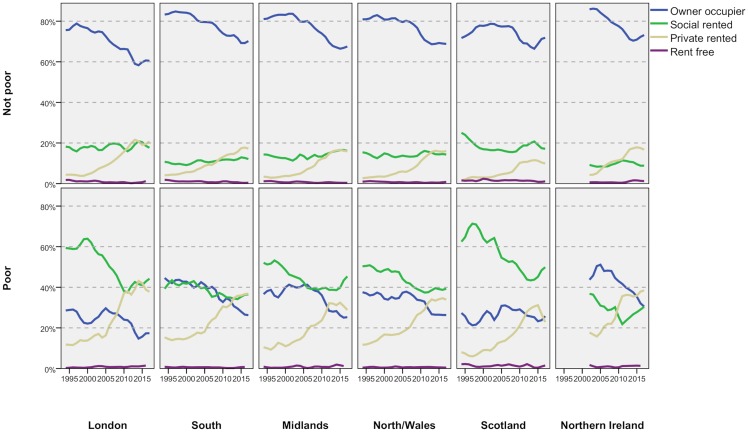
Regional variations in tenure by poverty status—Children, 1994/5-2017/8. FRS/HBAI data. Poverty defined as households below 60 per cent of contemporary median income, after housing costs. Years are financial years (i.e. ‘2017’ is 2017/18).

As with adults, there are substantial variations for children across the regions (Fig 9), with London having particularly high proportions of poor children in private renting: around 40 per cent in recent years which was as many as social renting. For the youngest children, a majority of those in poverty in the capital are in the PRS. Scotland has the lowest proportions of poor children in private renting due in large part to the greater role played by social rented housing there. The slowing in the growth of poor children in private renting in recent years is apparent in every region but there is also some slowing in the rise for non-poor children. In London, the latter group even declines slightly.

### Instability, private renting and poverty

**Fig 10:**
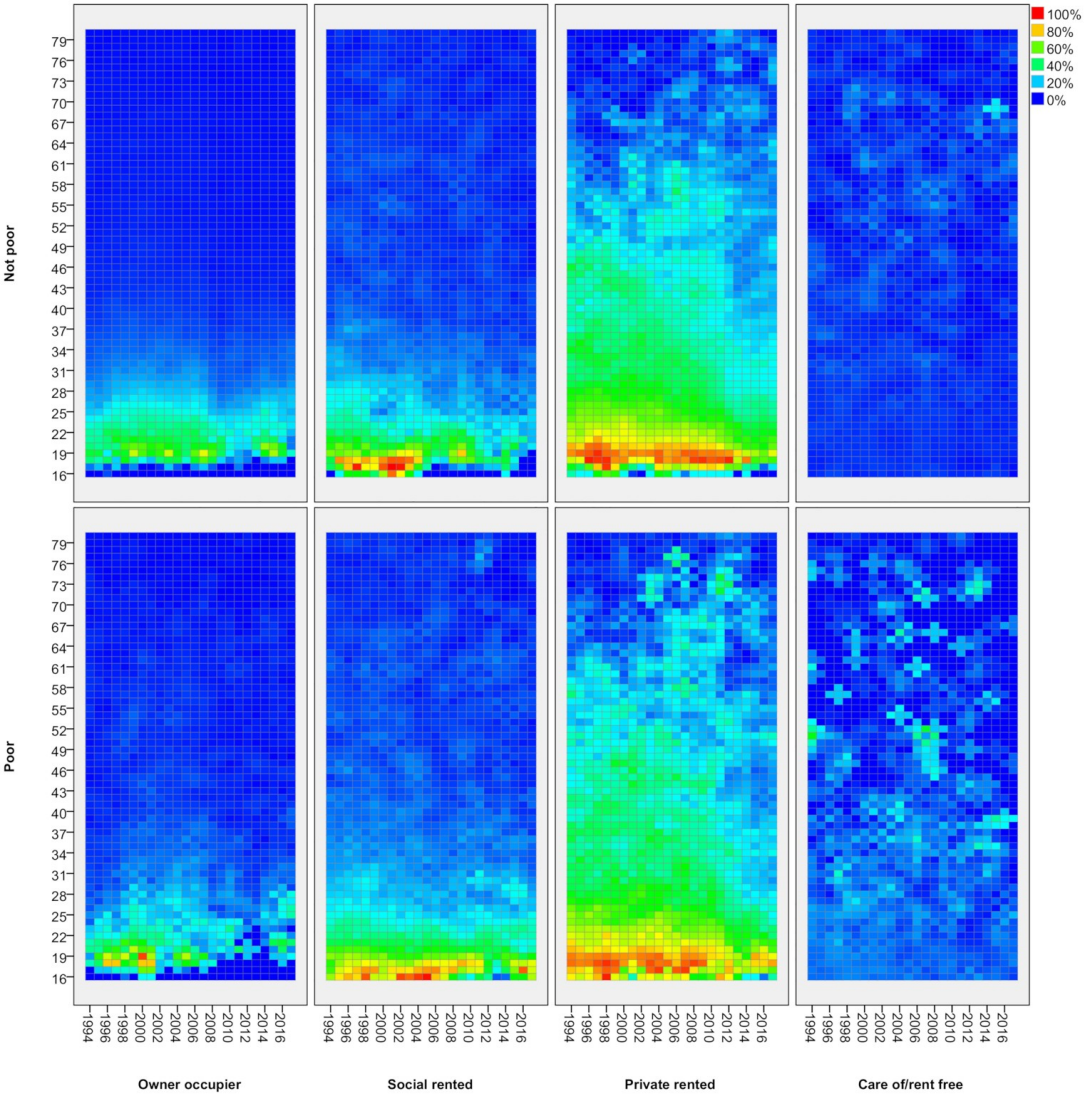
Proportions resident less than one year by tenure and poverty status—Adults, 1994/5-2017/8. FRS/HBAI data. Poverty defined as households below 60 per cent of contemporary median income, after housing costs. Years are financial years (i.e. ‘2017’ is 2017/18). In each cell, the colour represents the proportion of people for that age/year and poverty status who had been resident for less than one year in the property.

The last part of the analysis turns to the issue of security and stability of tenancies. The rise in poor households in the PRS would be less of an issue for policy if they were able to achieve security of tenure there. The question is therefore whether poor private renters face the same kinds of instability and short-term letting as others in the PRS or whether, on the contrary, a separate sub-sector of the PRS has emerged offering this group more stable, long-term rentals. As noted above, we do not have a measure of the legal security of tenure of PRS households but we can examine rates of mobility as some guide. Specifically, the FRS lets us examine the proportion of households who have been resident for less than one year in their current accommodation. This is an imperfect measure of insecurity since we cannot distinguish where recent moves reflect a positive choice or were a response to problems including forced relocation. Nevertheless, variations by age and poverty status, and over time give some insights into the nature of insecurity in different tenures (Fig 10).

For owner occupation and social renting, high levels of short residency only occur for people in their 20s/early 30s. At these ages, most people will have just entered these sectors. At later ages, mobility rates for these tenures are much lower as most people remain for many years in the same place. With the PRS, there are also high mobility rates for people in their early 20s associated with first entry to the sector but rates continue to be high for people in their 30s and 40s. There do not appear to be substantial differences between poor and non-poor adults for these two tenures. In both cases, mobility rates have fallen slightly, even allowing for the slight discontinuity in the data between 2011 and 2012 discussed above.

By contrast, high mobility in the PRS affects people well into their 30s and 40s, especially in the earlier years. The situation up to 2012 appears very similar for poor and non-poor adults. In the more recent years, mobility rates appear significantly lower in both cases, reflecting in part the discontinuity due to changes in question wording. In this period, mobility rates for poorer households do appear to be somewhat lower than for non-poor This may some indication that the sector is beginning to function a slightly different way for this group. For poor adults in their 30s, turnover may be higher than in social renting but it is lower than for non-poor adults of the same age. It is of course difficult to determine the extent to which this represents choice and satisfaction with current accommodation rather than constraint despite dissatisfaction.

## Conclusions and discussion

This paper shows that the ‘Generation Rent’ experience is not one shared equally by young people. While a number of existing studies have charted the uneven experience socially and spatially through qualitative work [44] [45], this paper contributes a much more detailed quantitative analysis which shows the scale and pace of the changes, and how the incidence varies with poverty status in particular. It makes clear therefore how the rise of private renting has been particularly marked for poorer young adults and their children. Private renting is now the dominant housing experience for this group. In 2017/18, 42 per cent of adults under 40 in poverty lived in private renting, almost double the proportion of 20 years ago, and more than owner occupation and social renting combined. For children in poverty, 33 per cent now live in private renting, three times the level of 20 years ago, and nearly as many as in social renting (42 per cent). For a substantial proportion of these children, this is likely to continue through primary and even secondary school years. Poor children are twice as likely to live in private renting as non-poor while poor adults are more than one and a half times as likely to do so. There are important regional variations, with the rises particularly large in the more pressured housing markets of London and the South of England, but they are found across the UK. The term ‘Generation Rent’ is thus a misnomer. As Coulter (2017) argued, there is a class divide here as much as a generational one.

The situation in the UK remains quite different to that in other liberal welfare regimes such as Australia or the US. Here, we still have a substantial social rented sector which continues to be an important destination tenure for working age adults. However it is also clear that a substantial, unplanned and largely unrecognised shift in the housing circumstances of poorer households has occurred over the last two decades towards a highly deregulated form of renting marked by problems of insecurity. Low-income groups face the same levels of insecurity in private renting as others but the impact of this is likely to be greater, both because it is likely to affect them for longer but also because it is likely to be a source of greater stress given their weak position in the housing market.

This shift in the housing circumstance of poor households can be seen as one manifestation of the broad retrenchment of welfare provision which has been underway in the UK as in many other countries in recent decades and which has coincided with rising insecurity in employment and earnings stagnation. In this particular case, it arises from the long-term reduction in provision of housing subsidies through social renting over the last four decades combined with the more recent reductions in personal housing subsidies (LHA) as well as reductions in wider welfare entitlement for the working age population, and young adults in particular. This makes young people increasingly dependent on family for their welfare [37], resulting in the rising inequality in housing experiences and outcomes we see here.

There is an urgent need to understand more about the welfare impacts of these changes, updating and extending Kemp’s [7] analysis. Building on studies which have begun to chart the social and spatial differences in the experience of contemporary private renting, including many cited in this paper, we need to understand the quality of housing provided to low-income private renters, both physical conditions and management, and the affordability of this tenure and its impacts on overall financial stress. We need to examine wider impacts, notably on employment incentives and outcomes, as well as access to social networks or support and the wider psycho-social impacts of insecurity. And we need to improve understanding of private renting careers, not just the length of individual tenancies.

The re-growth of private renting has been in line with the objectives of successive governments since the 1980s. However, the pace of change after 2000 clearly took government by surprise, leading to efforts to dampen down supply by increasing taxation on landlords, and reduce demand by introducing subsidies for first time buyers [28] [46]. There has been little recognition of how these changes impact on low-income households, let alone a proper debate about the reforms needed to ensure that group can access decent accommodation within this tenure. When combined with the restrictions on LHA entitlements, the recent changes appear to worsen the situation for these groups by making landlords less willing to house them.

The main policy change for private renting currently under discussion is a move to reintroduce security of tenure, in combination with limits on the ability of landlords to increase rents for sitting tenants. In theory at least, security of tenure has been reintroduced in Scotland for new tenancies from December 2017 and the UK Government has signalled an interest in a similar move for England and Wales. The consequences of such moves depend, on the one hand, on the effectiveness of the regulations and their enforcement and, on the other hand, on the responses of landlords in terms of investment decisions and their willingness to rent to low-income households. What is clear is that, on their own, these changes do not provide low-income households with any more resources for housing so they are likely to continue to struggle to find adequate accommodation in the market-driven PRS.

Finally, moving beyond the substantive discussion, the paper seeks to make an important methodological contribution by demonstrating the analytical value of Lexis surfaces. These are a powerful tool for rendering the growing quantities of social survey data accessible, particularly to non-technical audiences. In this case, they reveal how changes over time in one characteristic, housing tenure, have affected specific age groups at specific times without the need for modelling or other complex techniques. They enable these changes to be further disaggregated by a wide variety of categories, such as poverty status and region, or to be overlaid with other outcomes such as length of residence. In this case, these rich analytical descriptions reveal an example of a cohort change, captured by the term ‘Generation Rent’, but they are equally suited to revealing effects limited to particular age groups or occurring at particular points in time. As Minton [12] argues, they deserve to be used much more widely as part of the process of data visualisation and exploration ahead of and alongside theoretically-driven analyses.
